# The Serendipity of Viral Trans-Neuronal Specificity: More Than Meets the Eye

**DOI:** 10.3389/fncel.2021.720807

**Published:** 2021-10-04

**Authors:** Kevin Thomas Beier

**Affiliations:** Department of Physiology & Biophysics, University of California, Irvine, Irvine, CA, United States

**Keywords:** RABV, trans-synaptic, immune response, glial cells, cell-cell transmission, retrograde (backward) motion, rhabdovirus

## Abstract

Trans-neuronal viruses are frequently used as neuroanatomical tools for mapping neuronal circuits. Specifically, recombinant one-step rabies viruses (RABV) have been instrumental in the widespread application of viral circuit mapping, as these viruses have enabled labs to map the direct inputs onto defined cell populations. Within the neuroscience community, it is widely believed that RABV spreads directly between neurons *via* synaptic connections, a hypothesis based principally on two observations. First, the virus labels neurons in a pattern consistent with known anatomical connectivity. Second, few glial cells appear to be infected following RABV injections, despite the fact that glial cells are abundant in the brain. However, there is no direct evidence that RABV can actually be transmitted through synaptic connections. Here we review the immunosubversive mechanisms that are critical to RABV’s success for infiltration of the central nervous system (CNS). These include interfering with and ultimately killing migratory T cells while maintaining levels of interferon (IFN) signaling in the brain parenchyma. Finally, we critically evaluate studies that support or are against synaptically-restricted RABV transmission and the implications of viral-host immune responses for RABV transmission in the brain.

## Introduction

### Trans-Neuronal Viruses as Neuroanatomical Tools

Viruses are an integral component of the modern neuroscientist’s toolkit for neuroanatomical mapping. In particular, neurotropic viruses such as herpes simplex virus (HSV), pseudorabies virus (PRV), vesicular stomatitis virus (VSV), and RABV have been used to map the connectivity of pathways in the nervous system of multiple organisms. These viruses have numerous advantages over proteins and dyes for mapping connected pathways in the brain, including signal amplification, delivery of genetically-encoded effectors into defined cells, and the ability to transmit across multiple cells. We have described these advantages in detail elsewhere (Nassi et al., 2015; Beier, 2019; Rogers and Beier, 2021). The application of these viruses has enabled neuroscientists to map chains of connected neurons, facilitating analysis of connected pathways in the brain that are otherwise difficult to discern on a large scale using other methods. These collective advantages have led to the widespread adoption of viruses as valuable tools for circuit mapping and manipulation.

Perhaps most importantly, the spread of neurotropic viruses between connected cells is widely believed to occur through synaptic connections between neurons, and thus these viral vectors are typically referred to as “trans-synaptic tracers”. Synaptic specificity of viral transmission has been inferred due to the observation that more neurons express virally-expressed genes relative to non-neuronal cells after infection, and the pattern of viral transmission from neuron to neuron is roughly in accordance with our knowledge of neuroanatomy. It was thus concluded, without direct evidence, that viruses are transmitted between connected neurons through synaptic connections. If this scenario is correct, it is important to carefully consider what the mechanisms of synaptic-specific viral transmission are and how they contribute to the labeling patterns that we observe. Investigation of virus survival and how they manipulate host cells invariably involves interactions with the host immune system, which is designed to combat viral infections. Most viruses have evolved mechanisms to combat the host immune response and thereby enable the virus to spread and propagate to new hosts. These mechanisms are thus highly relevant to how neurotropic viruses transmit and label particular types of brain cells following infection.

In this review, we explore the mechanisms by which neurotropic viruses, principally RABV, evade the immune system to establish and maintain infection in the CNS. Effectively evading the host immune response in the periphery, and continuing to evade the innate immune system is critical for virulence, as the brain parenchyma can otherwise mount effective anti-viral responses. While it may appear that full immune evasion would be the most advantageous for RABV replication, RABV paradoxically stimulates a low-level of antiviral type I interferon (IFN) response, likely initiating in astrocytes. This low-level activation of IFN may benefit RABV by supporting both neuronal survival and immune cell death. We also highlight mechanisms by which IFN-producing cells are stimulated, and postulate that: (1) RABV and perhaps other neurotropic viruses may not transmit exclusively through synapses and instead regularly enter glial cells such as astrocytes; and (2) this lack of synaptic specificity has likely been selected for as a mechanism of eliciting low-level IFN response to evade immune cell mediated clearance. These observations have implications for the use of trans-neuronal viral vectors, including one-step RABV, as they complicate the interpretation that RABV spreads exclusively through neuronal synapses in the CNS.

### RABV Evades Immune System Control Primarily by Interfering With T Cell Migration

The success of viruses in transmitting from one host to another is dependent on the ability to evade the host’s anti-viral defenses. RABV typically transmits to the CNS following intramuscular infection, usually from a bite by an infected animal. RABV can be taken up directly by the terminals of motor neurons that project to the affected muscle or can be spread from the muscle to motor neurons, and can sequentially make its way along a chain of neurons *via* retrograde transmission from the spinal cord to the brain. Having reached the cranial nerves, it can spread to the salivary glands, where it is excreted in the saliva and can be transmitted to a new host. To achieve effective spread within the organism, RABV has evolved multiple mechanisms to escape detection and destruction by the innate immune system. For example, in mice infected with the highly virulent Challenge Virus Strain (CVS) of RABV, the majority of apoptotic cells were found to be leukocytes, not neurons (Baloul and Lafon, 2003), thereby demonstrating that RABV infection leads to the death of immune cells. This was also observed in humans after autopsy (Hemachudha et al., 2005; Tobiume et al., 2009), and macrophages, oligodendrocytes, and migratory T cells undergo apoptosis following natural RABV infection in humans (Adle-Biassette et al., 1996; Baloul and Lafon, 2003; Fernandes et al., 2011). This suggests that RABV can simultaneously combat the immune response while maintaining the integrity of the neurons that it infects, thereby limiting the ability of the immune system to detect and control RABV dissemination.

The ability to bypass T cell-mediated detection and control of infection is likely the key factor that first differentiates encephalitic from non-encephalitic RABV infection. This is supported by at least three lines of evidence. First, CVS RABV infection exhibited the same level of virulence in nude and control mice, implying that T cells do not effectively control CVS RABV infection (Lafon, 2005). Second, migrating CD3^+^ T cells became apoptotic after CVS RABV infection (Baloul and Lafon, 2003; Baloul et al., 2004), suggesting that they were ineffective in combatting RABV spread. Third, depletion of T cells, specifically CD4^+^ T cells, was sufficient to transform an abortive infection into an encephalitic infection, while depletion of CD8^+^ T cells had no effect (Iwasaki et al., 1977; Smith et al., 1982; Weiland et al., 1992; Xiang et al., 1995; Hooper et al., 1998; Galelli et al., 2000). In contrast, neurotropic viruses such as VSV that typically are not neuroinvasive are detected before they can access the CNS. For example, VSV is identified by macrophages in the subcapsular sinus, and when these cells were depleted, VSV became neuroinvasive and infections were lethal, which is similar to RABV (Iannacone et al., 2010). It is likely not the magnitude of the immune response that determines whether a neurotropic virus establishes an encephalitic infection or not, but rather that some viruses have evolved mechanisms to directly disable the immune response, as described for the CVS strain of RABV (Lafon, 2011).

After CNS infiltration, RABV must still actively interfere with the inflammatory response in order to evade detection, as migratory T cells are recruited to the site of CNS infection. Indeed, the extent to which RABV interferes with immune signaling is related to pathogenicity: more virulent RABV strains stimulate significantly lower levels of CC and CXC chemokines, complement factors, pro-inflammatory cytokines, and cytokine receptors than less virulent strains (Wang et al., 2005). As these receptors and ligands are critical for effective leukocyte recruitment to the brain, inhibiting their expression would result in reduced inflammation and less effective viral clearance. Leukocyte recruitment into the brain also requires the breakdown of the blood- brain barrier (BBB). Indeed, attenuated strains of RABV increase BBB permeability which leads to increased levels of CD4^+^ T cells and CD19^+^ B cells in the brain, while more virulent strains of RABV do not disrupt the BBB (Phares et al., 2006; Roy and Hooper, 2007). Notably, though T cells appear to be the major contributor to the defense against RABV, B cells may also play a role. For example, B cell entry into the CNS and antibody secretion is an important factor in viral clearance (Wunner et al., 1983; Wiktor et al., 1984; Montaño-Hirose et al., 1993; Hooper et al., 1998), and few B cells are seen in the brain of encephalitic infections (Camelo et al., 2000; Kojima et al., 2010), suggesting that preventing B cell migration is a key component of encephalitic infections.

### RABV Modulates IFN Signaling to Promote Neuron Survival

In addition to evading leukocytes, RABV must also maintain the integrity of infected neurons in order to facilitate viral spread and evade detection through viral components released by cell destruction. Clues as to how it achieves this goal can be found by comparing the consequences of infection with highly virulent vs. attenuated RABV strains. Relative to encephalitic infections, infections by attenuated strains that led to aborted infections resulted in many more apoptotic neurons, an effect likely mediated *via* neuronal destruction by T cells (Galelli et al., 2000; Baloul and Lafon, 2003). Consistent with this concept, neuronal apoptosis in RABV-infected animals was inversely correlated with RABV pathogenicity (Morimoto et al., 1998, 1999; Thoulouze et al., 2003), suggesting that preventing cellular apoptosis is a key strategy for RABV virulence. In order to preserve the viability of infected cells, RABV regulates the cellular antiviral response, the first line of which is mediated by type I IFNs which orchestrate the host response to viral infections. To identify foreign agents such as viruses or bacteria, components of foreign agents, known as pathogen-associated molecular patterns (PAMPs), are detected by pattern-recognition proteins (PRPs) including toll-like receptors (TLRs) in endosomes, and retinoic acid-inducible gene I (RIGI)-like receptors (RLRs) in the cytosol (Katze et al., 2008; Chow et al., 2018; Lee et al., 2019; Rehwinkel and Gack, 2020). Detection of PAMPs, in turn, elicits an antiviral response that includes activating interferon regulatory factor 1 (IRF-1), and driving expression of IFNα/β. IFNα/β secretion activates signaling *via* Type I IFN receptors (IFNAR) in adjacent cells. This IFN signaling is then key for eliciting a potent immune response and combatting encephalitic infections of neurotropic viruses. For example, Type I IFN signaling is required for mounting an effective immune response to VSV to prevent encephalitis (Detje et al., 2009; Drokhlyansky et al., 2017). While neurons are responsive to IFN, they do not appear to be the major producers of IFN in response to viral infections, though this appears to depend on several factors (Wang and Campbell, 2005; Delhaye et al., 2006; Yin et al., 2009; Kallfass et al., 2012).

Given that Type I IFNs stimulate an anti-viral defense program in the brain, actively combatting IFN is necessary for successful evasion of the host immune system. Indeed, the RABV N/P/M/G proteins all modulate the IFN response in infected cells (Lafon, 2011; Ito et al., 2016; Scott and Nel, 2016). Nonetheless, it is important to note that even highly virulent strains of RABV trigger a detectable IFN response in the host. In fact, the majority of differentially expressed genes in mice infected with RABV relative to mock-infected control mice were genes involved in the innate immune response and host defense mechanisms (Zhao et al., 2011). This response appears to be neuroprotective, as RABV displays increased virulence in IFNAR knockout mice that lack a functional IFN response (Chopy et al., 2011). By dampening but not completely eliminating IFN signaling, RABV prevents glial and immune cells from clearing the virus while triggering the expression of genes in neuronal cells that increase neuronal survival by inhibiting T cell recruitment. For example, RABV causes neuronal upregulation of HLA-G, FasL, and B7-H1 (Lafon, 2005, 2008; Mégret et al., 2005), proteins used to evade immune detection by triggering signaling pathways in T cells *via* CD8, Fas, and PD-1, respectively, that contribute to T cell death (Gratas et al., 1998; Dong et al., 2002; Rouas-Freiss et al., 2003). Virulence was reduced in mice lacking either FasL or B7-H1, proteins which are typically expressed in neurons, and thereby demonstrating that these proteins play critical roles in modulating virulence (Lafon et al., 2008). Notably, this strategy is also used by tumor cells to evade the immune response.

If stimulating low-levels of IFN signaling is neuroprotective and is a strategy used by RABV to promote neuronal survival and immune cell death, it is important to identify which cells produce Type I IFNs during RABV infections, particularly as RABV proteins interfere with IFN induction in RABV-infected cells. In addition, the question of whether neurons contribute to IFN production is somewhat controversial. In the absence of dedicated IFN-producing cells such as plasmacytoid dendritic cells, most brain cells including both neurons and glia have the capacity to contribute to IFN signaling, though the transient nature of IFN has made it difficult to conclusively determine which cells are principally responsible *in vivo* (Steinman, 1991; Detje et al., 2009; Sorgeloos et al., 2013; Owens et al., 2014). Multiple studies suggest that the most likely candidate is resident glial cells, principally astrocytes (Detje et al., 2015; Pfefferkorn et al., 2016; Drokhlyansky et al., 2017).

### Astrocytes Are the Major Source of Type I IFNs in the Brain Parenchyma

In order to contribute to IFN production, brain glial cells must either be infected with RABV or be exposed to RABV protein or RNA that would trigger an anti-viral response. Infection of glia is at face value inconsistent with viral circuit mapping studies, including the majority of those that employ one-step RABV, where few if any glial cells are labeled. If true, how can glial cells produce IFN to facilitate the survival of infected cells? The answer to this riddle may be that RABV does infect glial cells, but that the majority of such infections are aborted (Pfefferkorn et al., 2016). In a recent study, investigators generated a recombinant RABV expressing the Cre recombinase and injected it into the brain of a Cre reporter mouse that expressed tdTomato in cells with a Cre expression history. While the majority of cells expressing the RABV N-protein were neurons, there was a large population of cells that did not express detectable levels of RABV N but expressed tdTomato instead. The majority of these cells were astrocytes, as evidenced by overlap with the astrocyte marker glial fibrillary acidic protein (GFAP).

This study found that astrocytes but not neurons, microglia, or macrophages were the main producers of IFN-β in response to the direct intracranial infection of the neurotropic viruses Theiler’s murine encephalomyelitis virus (TMEV) or RABV, or intranasal infection using VSV or RABV. While technical limitations prevented definitive identification of abortively infected astrocytes as the major IFN-β producing cells, they did strongly suggest that astrocytes are abortively infected by RABV and that astrocytes were the primary cells to produce IFN. One significant limitation of this study is that they injected large volumes of virus (4 μl) into unspecified regions of the brain; therefore, it is not possible to discern if these glial cells were infected from the initial inoculum or transmission of the virus from the infected brain cells. Nonetheless, the study does indicate that astrocytes can be infected by RABV and that numerous such events lead to abortive infections that would not be observed in typical circuit mapping studies. Importantly, since glial cells were tdTomato^+^, this indicates that astrocytes are not only exposed to viral components but that virally-expressed Cre recombinase was transduced into these neurons, which strongly suggests that Cre protein was delivered by intact RABV virions. Notably, delivery of Cre packaged within the RABV virion is sufficient to enable recombination of infected cells, and thus it is not clear that viral replication in glia was necessary to induce recombination in astrocytes (Chatterjee et al., 2018).

### How Is “Trans-Synaptic Specificity” Achieved?

The principal evidence for the synaptic specificity of viral transmission is that neurotropic viruses including HSV, PRV, VSV, and RABV tend to label cells in a pattern consistent with expected neuroanatomical connectivity, as scored by expression of a virally-expressed protein in these cells. The study that is most often cited in support of trans-synaptic specificity was published by Gabrielle Ugolini, where RABV was used to map the circuits of facial motor control (Ugolini, 1995). In that study, RABV was injected in the hypoglossal nerve, and viral labeling was progressively observed in the medulla, midbrain, and cortex, in that order, a pattern consistent with known anatomical connectivity. However, this falls short of conclusively demonstrating synaptic specificity. While these results and those from many other subsequent studies are indeed consistent with the synaptic spread of the virus, they are also consistent with the spread of the virus to closely juxtaposed cells (Svoboda, 2019; Rogers and Beier, 2021). Viral spread from infected cells is not promiscuous, as supported by three lines of evidence. First, although RABV-infected cells are surrounded by processes from other neurons, which likely significantly outnumber the number of synaptic contacts made with other cells (Mishchenko et al., 2010; Kasthuri et al., 2015), RABV is not reported to spread to axons of passage. Second, while viral transmission may occur preferentially at sites that contain cellular machinery for viral egress and entry, cellular exocytosis takes place at both synaptic and non-synaptic sites (Patterson et al., 2010), yet the pattern of spread appears quite restricted. Third, glial cells ensheathe neuronal cells, including synapses (Eroglu and Barres, 2010), yet these cells are largely not infected, as evidenced by an absence of viral gene expression. So how is the observed restricted pattern of viral labeling obtained?

To understand the origin of the apparent synaptic specificity of viral transmission, we first need to consider the following: (1) Do viruses need to transmit through synapses? (2) Can viruses transmit through synapses? and (3) If so, do viruses transmit exclusively through synapses?

The first question is if viruses need synapses for intercellular transmission. To test this, one only needs to assess if RABV can transmit between cells that do not have neuronal synapses. Given that RABV can transmit in a variety of cell types in different organisms *in vivo* and tissue culture (Reagan and Wunner, 1985), synapses are not required for viral transmission. Furthermore, RABV can infect primary cultures of microglia and astrocytes of murine, feline, and human origin, and can replicate in at least a few of these cell types (Ray et al., 1997), demonstrating that RABV infection is not limited to neurons. Therefore, neuronal synapses are not required for viral infection/replication, and RABV can infect glial cells.

The second question is if viruses can transmit through neuronal synapses. To definitively demonstrate trans-synaptic transmission, one would need to observe virions exiting one neuronal cell and/or entering another neuronal cell at a synapse. In the case of RABV, this would likely be transmission from post-synaptic dendrites to pre-synaptic axons, in accordance with the retrograde trans-neuronal transmission. To our knowledge, no clear evidence for synaptic-specific spread of RABV has been reported. RABV has been observed transmitting between adjacent cells; active pinocytosis of virus in a secondary cell was observed before release from the primary cell (Iwasaki and Clark, 1975). This supports the possibility that viruses may transmit between processes of closely juxtaposed cells. However, in this same study, RABV was observed budding from multiple locations of the cell bodies of infected neurons, and it was not assessed whether the budding may occur specifically or preferentially at synaptic sites. Thus, in the absence of direct evidence of synaptic transmission of the virus, the answer to this question remains speculative at best.

The third question is if viruses transmit exclusively through synapses. This seems unlikely, as RABV virions were observed in the extracellular space surrounding neurons as well as budding from likely non-synaptic sites on neurons (Iwasaki et al., 1975). This demonstrates that not all viral releases occurred to pre-synaptic cells. It is therefore unclear if viruses can transmit through synapses, but non-synaptic transmission *in vivo* appears likely. In addition, one can assess whether RABV functionally transmits between cells in a non-synaptic manner by testing the fraction of infected cells that are synaptically connected to other virally-infected cells. The viral spread between non-connected neurons would be one line of evidence for non-synaptic-exclusive mode of transmission; the other would be infection of glial cells. In the original assessment of connectivity using one-step RABV, Wickersham and colleagues showed that 9 out of 11 RABV-labeled pairs were functionally connected, relative to 0 out of 9 nearby non-infected cells (Wickersham et al., 2007). Though this study was not comprehensive and was performed in cortical slice cultures which do not faithfully recapitulate connectivity in the living brain, they remain the best estimate for RABV’s preference for the spread between connected neurons. It is important to note that though these data suggest that RABV spread preferentially occurred between connected cells as assessed by cell labeling using a virally encoded protein, they fall short of showing that transmission occurs at synapses. Other explanations, such as that connected cells simply have closer appositions through which viruses could pass relative to non-connected cells, remain possible.

Therefore, viruses do not need to transmit through synapses, it is not clear if they even can, and they certainly do not do so exclusively. How then is the apparent specificity of labeling achieved?

### Abortive RABV Infection of Glia May Promote RABV Spread in the Brain

**Figure 1:**
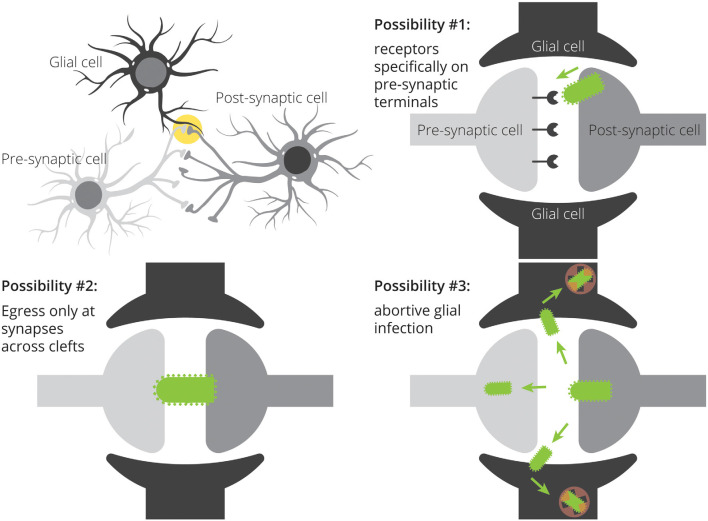
Three potential mechanisms for restricted patterns of RABV spread in the CNS. Spread restricted to preferentially synaptically-connected input cells could be achieved through specific expression of viral receptors on input cell terminals, direct transfer of viruses from post-synaptic cells to pre-synaptic cells without ever needing to bud into the extracellular space, and through non-productive infection of nearby glial cells. A combination of these mechanisms may also influence the apparent labeling specificity.

There are multiple ways by which viruses could transmit preferentially to neurons in close proximity. One is through the expression of viral receptors located only at particular sites on target neurons (Figure 1). This was originally postulated to be the mechanism of transmission of RABV: the nicotinic acetylcholine receptor (nAChR) was shown to be a receptor for RABV, and nAChRs are densely expressed at the neuromuscular junction (Lentz et al., 1982). As RABV infection of muscle tissue spatially coincided with expression of acetylcholinesterase, and injection of nAChR antagonists α-bungarotoxin and d-tubocurarine reduced the infection of myotubes, it was hypothesized that nAChRs mediate RABV infection. However, it was later shown that nAChRs are not necessary for RABV infection, as RABV could infect cells by other means (Reagan and Wunner, 1985). As of now, at least four putative RABV receptors have been identified: NAChR (Lentz et al., 1982), the low-affinity nerve growth factor receptor p75NTR (Tuffereau et al., 1998), neural cell adhesion molecule NCAM (Thoulouze et al., 1998), and the metabotropic glutamate receptor mGluR2 (Wang et al., 2018), yet none have been shown to be necessary for RABV infection. In the absence of a single receptor used by RABV with restricted expression to pre-synaptic terminals, restricted expression of viral receptors is unlikely to be the determining factor of transmission specificity.

**Figure 2:**
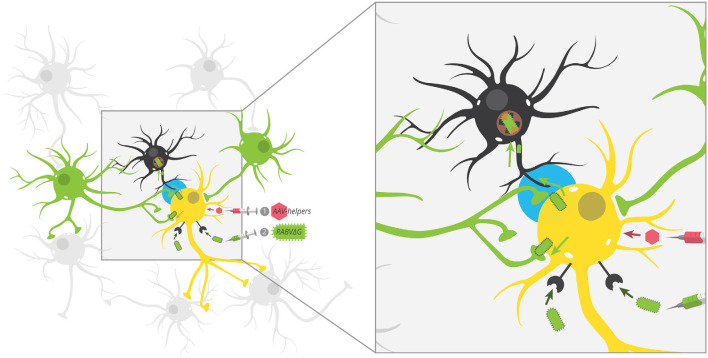
Potential unobserved glial infection using one-step RABV. AAV helpers expressing red fluorescent protein mark starter cells, which are then targeted by EnvA-pseudotyped RABV by cellular expression of TVA. Because RABV encodes GFP, this makes starter cells red + green = yellow. By trans-complementation, RABV can spread to direct input cells (green). According to our postulated model, RABV may also spread to nearby glial cells (black), but these infections are aborted and thus, glial cells don’t express GFP. This leaves the GFP label specifically in neurons that preferentially connect to starter neurons. The tripartite synapse is highlighted in blue.

The second possibility is that viruses transmit between closely juxtaposed sites where rapid endocytosis and exocytosis are taking place. The synapse is one such site. Furthermore, the synapse is connected to other parts of the cell by actin filaments and microtubules, and intact virions could reach dendrites and/or axons through rapid transport *via* kinesin and dynein molecular motors. Because chemical synapses span approximately ~20 nm from pre- to post-synaptic cells and the long axis of RABV is ~180 nm, this means that the virion is approximately nine times as large as the gap between pre- and post-synaptic cells. As RABV buds perpendicular to the cellular surface (Minamoto et al., 1978), this means that virions exiting a post-synaptic site would come into contact with a pre-synaptic cell long before it leaves the post-synaptic cell. Such direct cell-cell transmission is believed to be the primary mode of transmission of many viruses (Mothes et al., 2010) and does occur with RABV in neurons (Iwasaki and Clark, 1975). On the other hand, viruses do not need synapses to bud, and exocytosis also occurs at extra-synaptic sites (Patterson et al., 2010). Therefore, it is unlikely that viral transmission requires synapses *in vivo*. Further evidence in support of non-synapse-restricted transmission is that RABV virions were observed in the extracellular space between cells, demonstrating that virions can bud off from the primary cell without transmitting directly to another cell (Iwasaki et al., 1975). The third possibility is that glial cells do become infected with the virus, but they do not effectively express viral transgenes. If virions can bud from extra-synaptic sites, then glial cells should also become infected with the virus. Yet, the expression of RABV-encoded gene products is not typically observed in glial cells. However, evidence increasingly suggests that glial cells do become infected with RABV, though these often lead to abortive infections (Pfefferkorn et al., 2016). Furthermore, given that these cells are likely the source of IFN-β, it means that infection of glial cells-through non-synapse-specific-spread-is likely to promote survival of RABV-infected neurons. Since maintaining the health of infected cells appears to be a critical factor that determines virulence (Morimoto et al., 1999; Préhaud et al., 2003; Fu and Jackson, 2005; Sarmento et al., 2005; Dietzschold et al., 2008; Lafon, 2011), this mode of transmission would likely be positively selected over time. This means that, contrary to the hypothesis that RABV transmits directly between synaptically-connected cells in order to avoid glial cells and immune detection, RABV may spread to astrocytes (Figure 2), and this infection may be a way to increase the survival of infected neurons and ultimately increase the likelihood that the virus makes it to the salivary gland and ultimately new hosts. It could be that infecting astrocytes in order to stimulate low–levels of IFN signaling provides a selective advantage relative to a synapse-specific mode of transmission. Alternatively, it could be that complete synapse restriction of viral transmission is not possible and thus infecting astrocytes is unavoidable. In this case, modulating IFN signaling *via* abortively infected astrocytes may confer the greatest advantage given the constraint of non-synapse-specific spread.

### G-Deleted RABV That Cannot Spread Still Induces a Robust Immune Response

The majority of studies investigating the immune response elicited by RABV infection have used replication-competent strains of RABV. However, most neuroscientists prefer to use the single-step, G-deleted RABV. Given that this virus is typically administered directly into the brain, and spread is much more restricted than replication-competent versions, it is important to consider how findings using the replication-competent versions may relate to those using the G-deleted versions. A recent study from Huang and Sabatini injected a G-deleted RABV into one of four brain regions that receive innervation from the dorsal raphe (DR) and conducted single-cell sequencing experiments from the DR (Huang and Sabatini, 2020). They noted several observations that suggest that even RABV variants that cannot spread in the brain elicit robust immune responses. First, they detected about three times as many microglia, and about two hundred times as many lymphocytes and non-resident myeloid cells in the DR of animals infected with G-deleted RABV relative to non-infected controls, demonstrating that this virus can still elicit strong immune responses away from the site of viral injection, and can trigger infiltration of circulating leukocytes into the brain. Interestingly, the extent of leukocyte infiltration appeared to scale with the number of DR neurons infected with RABV. Second, infection with this G-deleted virus elicited a substantial upregulation of genes involved in the antiviral response, including MHC genes, genes involved in both type I and type II IFN responses, complement, as well as cytokines. These results were largely consistent with bulk RNA sequencing studies performed following infection with replication-competent RABV (Prosniak et al., 2001; Zhao et al., 2011, 2018), suggesting that the immune response is similar to either replication-competent or G-deleted variants. Third, the authors detected RABV transcripts in both neurons and non-neuronal cell types in the DR. Given that the virus should not be able to spread in the brain beyond initially-infected neurons and thus should only be present in DR cells that projected to the sites in the brain where RABV was injected, RABV genes should only be expressed in neurons. The authors hypothesized that the presence of RABV transcripts in other cells may be due to phagocytic capture of RABV transcripts. Notably, the level of RABV transcripts in these cells was significantly lower than in neuronal cells. While such a phagocytic capture is possible, in that case, it is likely that other neuronal genes should be observed in these phagocytic cells, though this was not reported. Therefore, another hypothesis is that phagocytic cells can phagocytose G-deleted RABV particles released from RABV-infected cells, and these virions then exhibit minimal replication capacity in these cells. It has been shown that RABV particles can bud from infected cells that do not express G, albeit at reduced efficiency (Mebatsion et al., 1996). Therefore, this result is entirely consistent with a potential abortive infection of glial cells by RABV, whether the RABV contains G on the surface or not.

### Currently Used Viruses for Circuit Mapping Applications Elicit Immune Responses

Here we have largely focused on the immune response triggered by RABV and mechanisms used by RABV to evade the immune system. However, other viruses such as VSV, PRV, and HSV are also in use as viral circuit tracers, and all have evolved different mechanisms to interact with and evade the host immune response, to greater or lesser effect. The manner in which the viruses interact with the immune system plays an important role in the viral life cycle, their host interactions, and the degree of neuroinvasiveness. For example, VSV is not typically neuroinvasive when administered peripherally, as it gets detected and stopped by subcapsular macrophages before entering the central nervous system (Iannacone et al., 2010). When administered intranasally, the IFN response elicited is sufficient to prevent substantial neuroinvasion that would cause a lethal infection (Detje et al., 2009). Interestingly, the extent to which PRV activates the immune system appears to be related to its neurovirulence; for example, when administered peripherally, the PRV-Becker strain elicits a robust immune response and demonstrates only weak neuroinvasiveness, while the less pathogenetic PRV-Bartha strain elicits a much weaker immune response and robust neuroinvasion (Brittle et al., 2004; Brukman and Enquist, 2006). HSV, like RABV, can be neuroinvasive, and trigger a robust immune response both peripherally and centrally. Unlike RABV, HSV has both a lytic and latent phase, which requires a dynamic interplay between the anti-viral defense mechanisms and the virus itself. For example, while IFN is responsible for regulating HSV spread in neuronal and non-neuronal cells, the downregulation of IFN is critical to establishing lifelong latency, which typically occurs in sensory neurons (Rosato and Leib, 2015). The mechanisms of immune system activation in response to HSV have been reviewed extensively elsewhere (Chew et al., 2009; Conrady et al., 2011; Paludan et al., 2011; Ike et al., 2020). While the exact effects of the immune response on transneuronal mapping experiments using VSV, PRV, and HSV have not been studied in detail, that an immune response is elicited in the brain following neuroinvasion by each of these viruses suggests that many of the same concerns that apply to RABV, also apply to other viruses. We suspect that the immune system may influence the efficiency of transmission of each of these viruses in the brain, but further study is needed to test if this occurs, and how the immune system may influence viral spread.

### Conclusion and Future Considerations

Discussions surrounding the purported synaptic specificity of RABV must consider the potential consequences of abortive viral infection of glia. It may be that RABV spread to glial cells, and therefore, RABV transmission, occurs selectively at synapses, though not *via* canonical neuron-neuron pre-post synaptic connections. However, this seems unlikely, given that the majority of abortively infected glial cells are found in white matter, suggesting that virus is released from infected cell processes in the absence of synapses. The principal evidence in support of synapse specificity of RABV transmission is that viral labeling roughly recapitulates expected connectivity patterns, and in the original study that tested the connectivity of virally-infected cells, these pairs appear to be connected well above chance (Wickersham et al., 2007). The principal evidence against synapse specificity is the presence of RABV virions budding from non-synaptic sites, and the abortive infection of glial cells (Iwasaki and Clark, 1975; Pfefferkorn et al., 2016), though it is again important to note that it is unclear whether the abortive infection of glial cells was initiated by the initial inoculum or viral spread from infected brain cells. One could reconcile these observations by considering that perhaps *the means used to achieve the end don’t matter—the majority of cells expressing virally-mediated genes do happen to be connected to one another*. Perhaps viruses do not transmit through synapses, but the transmission events that lead to functional infection events preferentially occur between connected cells, and those that occur between non-connected cells, or to glia, often do not produce production infections.

Therefore, from a neuroanatomical perspective, the observed patterns of RABV labeling chains of neurons in a configuration consistent with the known anatomical connections may be a serendipitous convergence of several factors. First, if RABV infections of astrocytes are typically aborted rather than successful, this may trigger low/transient levels of IFN signaling that promote neuronal health and combat the actions of migratory T cells. This would restrict virally-mediated gene expression in astrocytes, resulting in the appearance of neuron-specific transmission of the virus. Second, because RABV promotes neuronal survival, it prevents non-selective virus release through apoptosis or necrosis. Third, the endocytosis/exocytosis machinery that facilitates successful viral infections may be concentrated near synapses, reducing promiscuous labeling of nearby cells, though this hypothesis has not been tested. Further experiments should be performed to more carefully address these possibilities. For example, a rigorous quantification of neurons and astrocytes labeled using one-step RABV variants encoding a fluorescent protein relative to variants expressing Cre in a Cre reporter mouse would help test if abortive astrocyte labeling is a general phenomenon, if other glial cells are labeled, and when this occurs during the course of circuit mapping experiments.

In spite of these limitations, we do not discourage the use of RABV as a trans-neuronal tracer—indeed we have used it successfully to map neuronal pathways that contribute to normal and pathological behaviors (Beier et al., 2015, 2017, 2019; Lerner et al., 2015; Schwarz et al., 2015; Weber et al., 2015, 2018; Chung et al., 2017; François et al., 2017; Cardozo Pinto et al., 2019; Choi et al., 2019; Holt et al., 2019; Wall et al., 2019; Steinberg et al., 2020; Shuster et al., 2021). However, the lack of direct evidence supporting the model of elegant synapse-specific viral transfer necessitates careful consideration of how to employ RABV circuit mapping in different contexts. For example, it is not clear if and how labeling preferences differ between different cell types or connections. Even though the virus may preferentially label connected cells, the manner by which it achieves this may be a consequence of several factors related to the modulation of cellular signaling pathways that prevent immune detection. After all, though we use RABV to map circuits, RABV evolution is based on selective pressure to increase the likelihood of spreading to new hosts. Elucidation of the detailed cellular mechanisms by which RABV has achieved synaptic specificity—or not—would facilitate understanding of how neurotropic viruses transmit in the brain, how to build better trans-neuronal tracers to map circuit organization, and provide critical information about the topology of cellular interactions more generally in the CNS.

## Author Contributions

KTB wrote the manuscript.

## Conflict of Interest

The author declares that the research was conducted in the absence of any commercial or financial relationships that could be construed as a potential conflict of interest.

## Publisher’s Note

All claims expressed in this article are solely those of the authors and do not necessarily represent those of their affiliated organizations, or those of the publisher, the editors and the reviewers. Any product that may be evaluated in this article, or claim that may be made by its manufacturer, is not guaranteed or endorsed by the publisher.
